# Exposure to androgen deprivation therapy and risk of anastomotic leakage after colorectal cancer surgery

**DOI:** 10.1111/codi.70126

**Published:** 2025-06-03

**Authors:** Martin Rutegård, Isac Norrgård, John Moshtaghi‐Svensson, Jaana Hagström, Ida Hed Myrberg, Anna Lantz, Suvi Rasilainen, Caroline Nordenvall, Malin Sund

**Affiliations:** ^1^ Department of Diagnostics and Intervention, Surgery Umeå University Umeå Sweden; ^2^ Department of Surgery University of Helsinki and Helsinki University Hospital Helsinki Finland; ^3^ Clinical Epidemiology Division, Department of Medicine Solna Karolinska Institutet Stockholm Sweden; ^4^ Department of Oral Pathology and Radiology University of Turku and Turku University Hospital Turku Finland; ^5^ Department of Pathology Helsinki University and Helsinki University Hospital Helsinki Finland; ^6^ Translational Cancer Research Program Unit University of Helsinki Helsinki Finland; ^7^ Department of Molecular Medicine and Surgery Karolinska Institutet Stockholm Sweden; ^8^ Department of Medical Epidemiology and Biostatistics Karolinska Institutet Stockholm Sweden; ^9^ Department of Pelvic Cancer Colorectal Surgery Unit, Karolinska University Hospital Stockholm Sweden

**Keywords:** anastomosis, hormones, leak, men, operation, receptor

## Abstract

**Aim:**

The risk of anastomotic leakage after colorectal cancer surgery is higher in men, regardless of the anatomical location. Previous studies suggest that this might be due to hormonal differences. The aim of this work was to investigate whether androgen deprivation therapy influenced the incidence of anastomotic leakage.

**Method:**

This is a nationwide registry‐based study of men operated on between 2007 and 2021 for colorectal cancer with an anastomosis. Exposure to androgen deprivation therapy (prescribed drugs or surgical castration) was related to anastomotic leakage using mixed‐effects logistic regression models. Two control groups were formed: one without and one with prostate cancer but without androgen deprivation. To study the potential target for androgen effect in intestinal tissue, androgen receptor expression was evaluated using immunohistochemistry in a smaller independent cohort to compare receptor expression in relation to leakage.

**Results:**

Some 24 611 men were included in the registry study, of whom 2.4% were exposed to androgen deprivation therapy. In this exposed group, compared with unexposed men with and without prostate cancer, respectively, leak rates were 3.7%, 5.6% and 5.8%, respectively. After adjustment, a nonsignificant reduction of anastomotic leakage in the exposed group was detected (OR 0.70, 95% CI 0.45–1.09) compared with men without prostate cancer. Tissue expression of androgen receptor was very low among patients with and without leakage, albeit with a trend of higher expression among the latter.

**Conclusion:**

Anastomotic leakage rates might be lower in men exposed to androgen deprivation therapy before surgery for colorectal cancer, although this finding must be interpreted cautiously. Effects on anastomotic healing do not seem to be mediated through classical androgen receptor signalling in the intestine.

**What does this paper add to the literature?**

The occurrence of anastomotic leakage after surgery for colorectal cancer differs between men and women, for unknown reasons. Here, nationwide registry data are used to demonstrate that men exposed to androgen deprivation therapy might have a lower rate of leakage. However, this potential effect does not seem to be mediated through colonic androgen receptors.

## INTRODUCTION

Anastomotic leakage after colorectal cancer surgery is a major complication, with associated morbidity and, at times, mortality [1, 2]. Technical as well as biological factors are involved in the development of leakage, and a strong link has been demonstrated between a distal anastomosis and male sex [1, 3]; in particular, men seem to have a 50% higher incidence of anastomotic leakage. A popular explanation for this discrepancy regarding rectal cancer is the wider pelvis in women, enabling an easier and less time‐consuming dissection; there is some support for this notion, as these sex differences are attenuated when taking pelvimetric measurements into account [4]. However, even colon cancer operations exhibit higher leak rates in men, although that difference might be explained by dissimilarities in visceral fat distribution [5].

In other biological processes, hormonal differences between men and women are essential, exemplified by improved skin healing via antiandrogen and oestrogen exposure [6]. The data on colorectal anastomoses are sparse, but experimental research implies that, in male mice, less collagen is deposited in colonic anastomoses [7]. In a previous population‐based study evaluating oestrogen exposure in women, hormonal replacement therapy seemed to decrease anastomotic leakage [8]. Previously, local prostate cancer treatment has been associated with an increased proportion of anastomotic leaks in rectal cancer patients [9]. However, the potential impact of antihormonal therapy remains unexamined, and the underlying mechanism by which androgens may influence anastomotic healing has yet to be explored.

In the present study, it was hypothesized that exposure to androgen deprivation therapy in men might decrease the risk of colorectal anastomotic leakage in comparison with nonexposed men with and without prostate cancer. Furthermore, it was hypothesized that there might be differences in androgen receptor expression in the intestinal tissue between patients with and without an anastomotic leakage, potentially providing insight into the signalling pathways involved.

## METHOD

### Checklist for the reporting of observational studies

This article was written in accordance with the Strengthening the Reporting of Observational Studies in Epidemiology (STROBE) checklist for the reporting of observational studies [10].

### Data source

A nationwide registry study was performed based on Colorectal Cancer Base Sweden (CRCBaSe), a database linkage originating from a national quality registry, the Swedish Colorectal Cancer Registry (SCRCR), containing data on all Swedish patients diagnosed with colorectal cancer [11]. Compliance has been assessed at 98.5% and 98.8% for colon and rectal tumours, respectively [12]. Data are prospectively registered during treatment and follow‐up. Patient and tumour characteristics such as age, sex, American Society of Anesthesiologists grade, tumour location and tumour stage are reported in detail, as well as preoperative treatment and perioperative data. Several other registries are incorporated into CRCBaSe. The Prescribed Drug Registry reports information on all prescribed and dispensed drugs since 2005 [13]. This registry contains information on Anatomical Therapeutic Chemical (ATC) code, dosage and date of prescription as well as withdrawals. The National Patient Registry includes dates and codes of diagnosis and surgical procedures. Data on inpatient care has been collected since 1964 (with nationwide coverage since 1987) and outpatient data since 2001, with a national coverage of more than 99% [14]. Information regarding educational attainment was obtained from the Longitudinal Integrated Database for Health Insurance and Labour Market Studies (LISA) database [15]. The Registry of Total Population provided information on migration [16] and the Cause of Death Registry was used to ascertain vital status.

### Study design

This is a retrospective observational study comprising men diagnosed with colorectal cancer between 2007 and 2021, identified in the SCRCR. The inclusion criteria were colorectal cancer surgery with an anastomosis; the exclusion criteria amounted to patients registered with procedural codes for prostatectomy or prostate irradiation without a concurrent prostate cancer diagnosis. The SCRCR was used to derive clinical data regarding tumour specifics, surgery and oncological treatment, whereas information on concurrent disease and educational attainment was collected from the National Patient Registry and the LISA database, respectively.

### Study exposure and outcome

#### Exposure

The exposure was classified according to ATC codes derived from the Prescribed Drugs Registry and diagnostic codes from the National Patient Registry. The exposure was androgen deprivation therapy (ADT). This was either due to antiandrogen therapy or chemical or surgical castration in men with prostate cancer. To be classified into this group due to drug exposure, the relevant drugs had to be withdrawn at least twice consecutively, at least one time of which was within 12 months before the colorectal cancer operation. Antiandrogen drugs comprised agents from the ATC group L02BB, while chemical castration consisted of drugs from the following ATC groups: L02AE02, L02AE03, L02AE04 or L02BX02. Surgical castration was determined by previous bilateral orchidectomy, as indicated by the occurrence of any procedural code KFC10 or KFC15 in the National Patient Registry before the colorectal cancer operation. Patients simultaneously fulfilling criteria for the antiandrogen group and castration group were excluded from the former but included in the latter group. The unexposed men consisted of men with prostate cancer without ADT, or men without prostate cancer.

#### Outcome

The primary outcome was anastomotic leakage within 30 days or in‐hospital. The SCRCR does not provide a formal definition of leakage. The variable has been evaluated for rectal cancer surgery and found to be underreported (29%) when compared with an international consensus definition [17], whereas almost no false positives could be found [18]. Moreover, the SCRCR does not include date and modality of detection for anastomotic leakage.

#### Tumour location

Right‐sided cancers were defined as any adenocarcinoma from the caecum to the transverse colon; left‐sided cancers included the splenic flexure down to the sigmoid; rectal cancers denoted any cancer within 15 cm from the anal verge, as measured by a rigid sigmoidoscope.

### Statistical analyses

**FIGURE 1:**
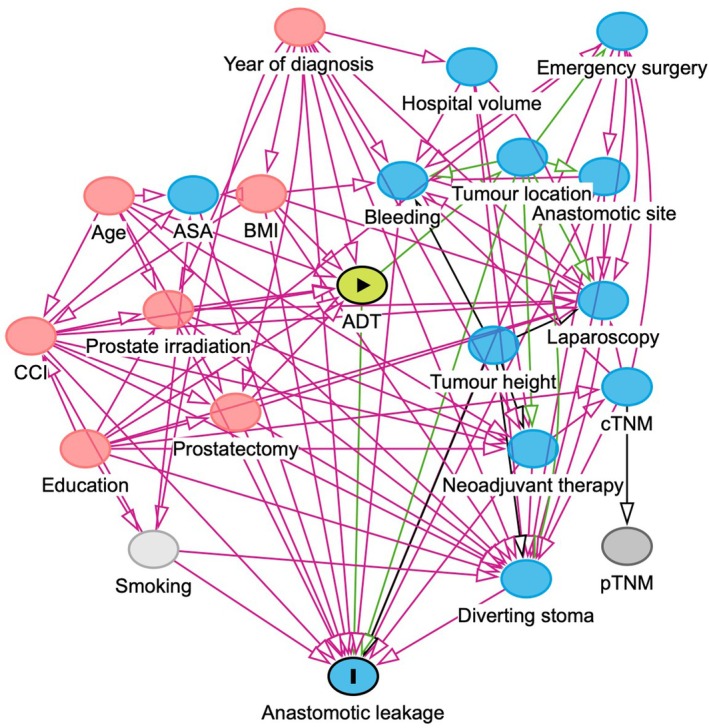
Directed acyclic graph, depicting the proposed relationships between exposure, outcome and other variables pertaining to the research question. Red circles indicate ancestors of both outcome and exposure that are necessary to adjust for to eliminate confounding. ADT, androgen deprivation therapy; ASA, American Society of Anesthesiologists physical fitness grade; BMI, body mass index; CCI, Charlson comorbidity index; c/pTNM, clinical/pathological tumour‐node‐metastasis staging system.

We aimed to estimate the total effect of ADT on the risk of anastomotic leakage, which requires adjustment for confounding. Potential confounding factors were chosen from a directed acyclic graph [19], depicted in Figure 1. A minimally sufficient adjustment set was defined, comprising age at diagnosis, calendar year of surgery, body mass index (BMI), Charlson comorbidity index (CCI) [20] and educational attainment; the first three variables were included as continuous variables, while BMI and education were treated as categorical variables (<25, 25–30 or >30 kg/m^2^ and <9, 9–12 or >12 years, respectively). When deriving the CCI, the prostate cancer diagnosis itself was not included in this study, as this formed part of the exposure. Unadjusted logistic regression as well as mixed‐effects logistic regression analyses with adjustment for the above covariates as fixed effects and a random intercept for the operating hospital were conducted, producing ORs with 95% CIs. Operating hospital was included as a cluster variable, since patients were extracted from all hospitals performing colorectal cancer resections during the study period. To assess multicollinearity of the mixed model, generalized inflation variance factors were determined for the main model; as these were close to unity for all predictors, the risk of multicollinearity was considered negligible. Moreover, an interaction model was used to estimate separate effects of ADT according to different tumour locations, employing an interaction term of ADT and tumour location. Sensitivity analyses were also conducted. Firstly, we evaluated the impact of including previous prostatectomy and/or prostate irradiation as confounders. Secondly, we redefined the ADT exposure to reflect a more stringent definition, requiring two dispensations within 24 months, of which one was within 12 months of surgery. Thirdly, we included ASA fitness grade (I, II or III–IV) as an additional covariate. A complete cases analysis was used throughout, as missing data were rare. The statistical analyses were performed in R version 4.1.1.

### Analysis of androgen receptor expression in intestinal tissue: A substudy

In this substudy, we used immunohistochemistry to compare the expression of androgen receptor in colonic tissue between patients who had experienced anastomotic leakage and patients who had not. An independent cohort, unrelated to the cohort used in the nationwide registry‐based study, was used for this purpose. Tissue samples were available for this cohort that included patients from previously published clinical trials [21, 22]. All patients underwent colon resection with an anastomosis between 2010 and 2019 at Helsinki University Hospital in Finland, and tissue samples were acquired from the Department of Pathology at the same hospital. A case–control matching was carried out, with cases being patients who experienced anastomotic leakage while controls did not. The tissue samples were selected from the healthy, proximal resection margin. Only colonic tissue was used, and patients whose tissue was of insufficient quality and with signs of malignant tissue were excluded.

#### Immunohistochemical staining

Immunohistochemical staining was carried out following the EnVision FLEX High pH detection system protocol (K8000, Dako, Glostrup, Denmark), utilizing a PT link (Dako, Glostrup, Denmark) and Autostainer 480S (Lab Vision, Fremont, CA, USA). Formalin‐fixed paraffin‐embedded tissue samples were cut to 3.5 μm sections. Deparaffinization, rehydration and antigen retrieval via heat‐induced epitope retrieval were conducted following the three‐in‐one specimen preparation procedure protocol (Dako, Glostrup, Denmark) using the Envision FLEX Target Retrieval Solution High pH (×50). This was followed by blocking of endogenous peroxidases using the Envision FLEX peroxidase‐blocking reagent (15‐min incubation). The primary antibody used was 0.22 mg/mL androgen receptor (AR) rabbit monoclonal antibody (SP107, Invitrogen, CA, USA; diluted 1:200, overnight incubation). EnVision FLEX/HRP was used as a secondary antibody (20‐min incubation). The staining was visualized using diaminobenzidine (10‐min incubation) as a chromogen and Mayer's haematoxylin as a counterstain. Sections of prostate gland tissue were used as positive controls during the staining.

#### Scoring of immunohistochemical staining

A binary scoring system (positive/negative) was used for assessing the immunohistochemical staining. The epithelium and the muscularis propria were scored separately. The expression in the respective tissues was considered positive if any immunopositivity was present and negative if no immunopositivity could be observed. The scoring was carried out by two evaluators (IN and JH), one of whom is an experienced clinical pathologist.

#### Statistical analysis of tissue staining

The scoring of the immunohistochemical staining was analysed and compared between the case and control groups using a nonparametric McNemar test. The expression in the epithelium and the muscularis propria was analysed separately. Statistical analysis was carried out using SPSS version 29.

## RESULTS

### Study participants

**FIGURE 2:**
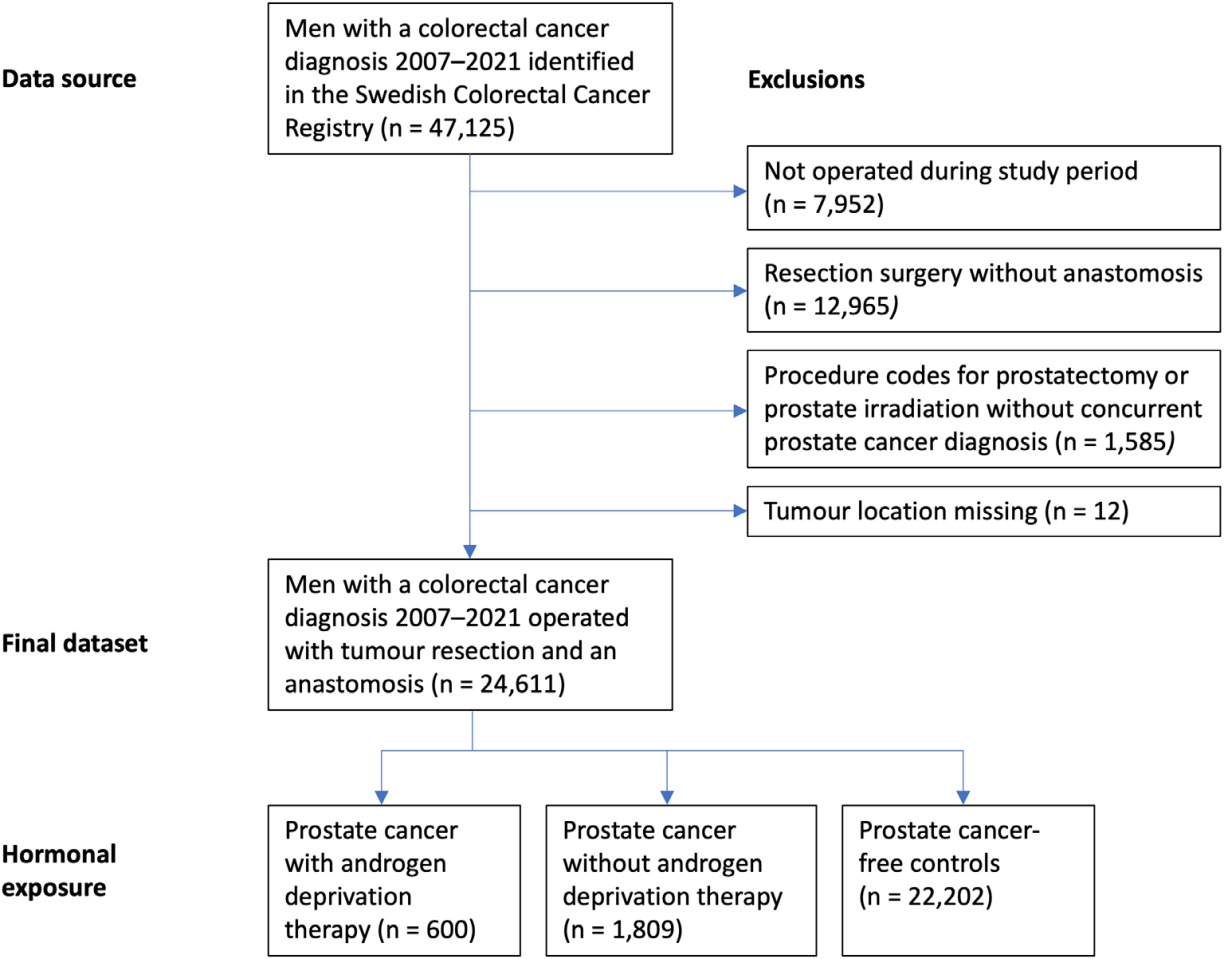
Flowchart for the nationwide registry‐based study.

**FIGURE 3:**
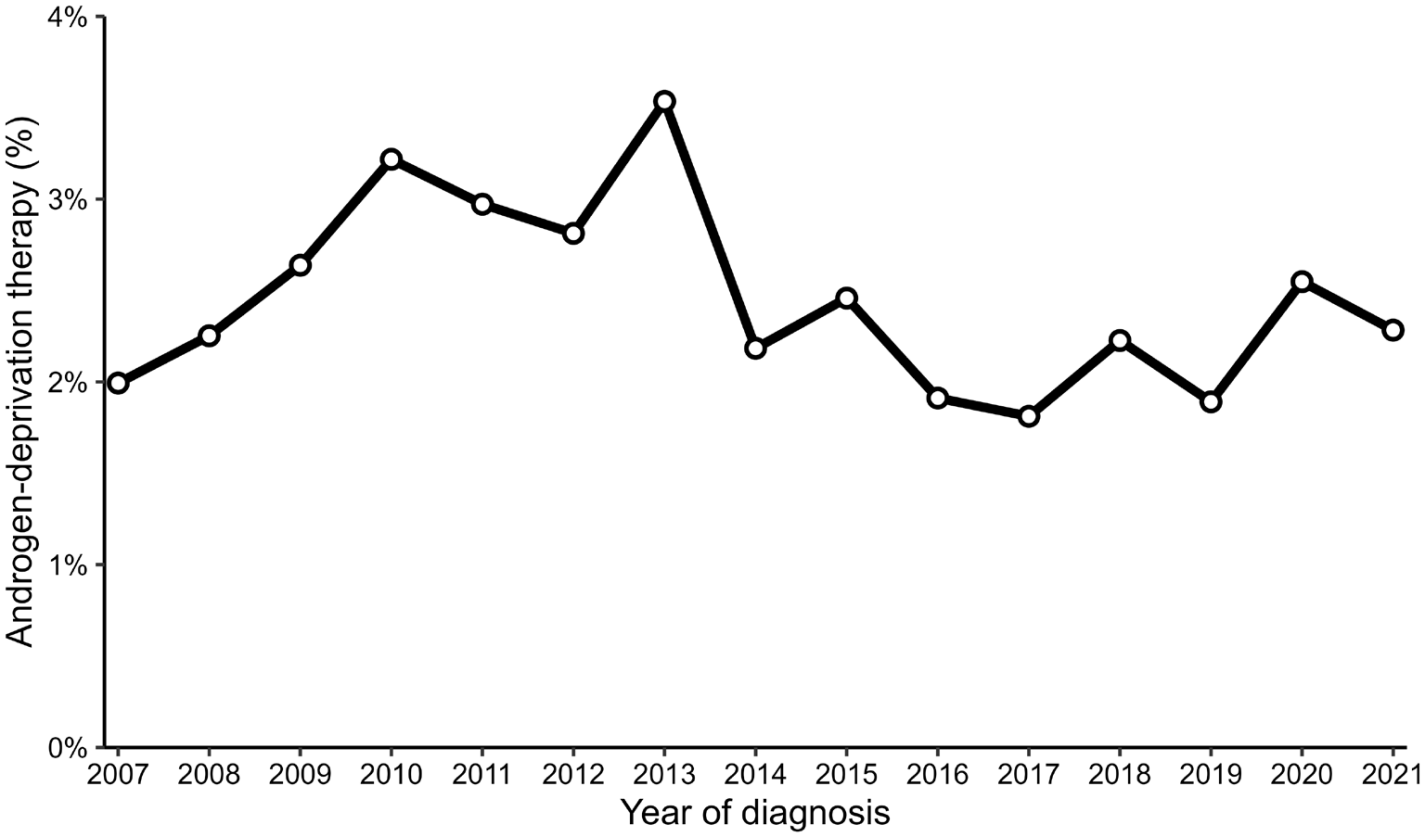
The yearly proportion of colorectal cancer patients receiving androgen deprivation therapy.

Some 24 611 men were included in the nationwide registry‐based study. Of these, 600 (2.4%) had prostate cancer and were considered exposed to ADT, 1809 (7.4%) had prostate cancer without ADT exposure, while 22 202 (90.2%) had no diagnosis of prostate cancer (Table 1, Figure 2). A minority of the ADT exposure group consisted of surgically or chemically castrated men (37.8%), while the remainder comprised men exposed to antiandrogens. The ADT exposure was consistent over time (Figure 3). Of note, those exposed to ADT were older, had fewer years of education, less commonly had a rectal cancer diagnosis and had a higher postoperative mortality.

**TABLE 1:** Demographic and clinical data for 24 611 men operated on in Sweden for colorectal cancer between 2007 and 2021, divided by prevalence of prostate cancer and androgen deprivation therapy.

Variables	Men without prostate cancer (*n* = 22 202)	Men with prostate cancer and ADT (*n* = 600)	Men with prostate cancer without ADT (*n* = 1809)
Age (years)	71 (64–78)	80 (75–84)	75 (70–79)
Body mass index (kg/m^2^)			
<25	8154 (37%)	237 (40%)	710 (39%)
25–30	8826 (40%)	227 (38%)	757 (42%)
>30	3524 (16%)	85 (14%)	244 (13%)
Missing	1698 (8%)	51 (8%)	98 (5%)
CCI^a^	0 (0–1)	0 (0–2)	0 (0–1)
ASA fitness grade			
I	3221 (15%)	12 (2%)	115 (6%)
II	11401 (51%)	263 (44%)	1031 (57%)
III–IV	7155 (32%)	306 (51%)	635 (35%)
Formal education (years)			
<9	7636 (34%)	258 (43%)	600 (33%)
9–12	8929 (40%)	225 (38%)	689 (38%)
>12	5387 (24%)	112 (19%)	502 (28%)
Prostatectomy	N/A	54 (9%)	549 (30%)
Prostate irradiation	N/A	165 (28%)	376 (21%)
Year of colorectal cancer diagnosis	2014 (2010–2018)	2013 (2010–2017)	2015 (2011–2019)
Tumour location			
Right colon	10346 (47%)	320 (53%)	895 (49%)
Left colon	8175 (37%)	217 (36%)	602 (33%)
Rectum	3681 (17%)	63 (10%)	312 (17%)
Pathological tumour stage			
0	56 (0%)	3 (0%)	5 (0%)
I	4096 (18%)	118 (20%)	371 (21%)
II	7541 (34%)	218 (36%)	611 (34%)
III	7619 (34%)	183 (30%)	596 (33%)
IV	2221 (10%)	58 (10%)	161 (9%)
Missing	669 (3%)	20 (3%)	65 (4%)
Emergency surgery	2566 (12%)	86 (14%)	153 (8%)
Minimally invasive surgery	6699 (30%)	173 (29%)	626 (35%)
Mortality within 90 days	763 (3%)	40 (7%)	60 (3%)

*Note*: Data are presented as median (IQR) for continuous measures and *n* (%) for categorical measures. Percentages may not add up due to rounding.

Abbreviations: ADT, androgen deprivation therapy; ASA, American Society of Anesthesiologists; CCI, Charlson comorbidity index.

^a^ Prostate cancer diagnosis excluded from the index calculation.

### Anastomotic leakage by ADT exposure

Men without a prostate cancer diagnosis had an overall leakage rate of 5.8%, while ADT‐exposed prostate cancer patients had a leakage rate of 3.7% and men with prostate cancer without ADT‐exposure had a rate of 5.6% (Table 2). Reintervention for leakage in these groups amounted to 73.5%, 77.3% and 69.6%, respectively.

**TABLE 2:** Anastomotic leakage as a function of prostate cancer prevalence and androgen deprivation therapy exposure in men operated for colorectal cancer, as well as by tumour location using an interaction term.

ADT group	Leakage proportion	Unadjusted OR (95% CI)	Adjusted^a^ OR (95% CI)
All locations			
Men without prostate cancer	1292/22,202 (5.8%)	Reference	Reference
Men with prostate cancer and ADT	22/600 (3.7%)	0.62 (0.39–0.92)	0.70 (0.45–1.09)
Men with prostate cancer without ADT	102/1,809 (5.6%)	0.97 (0.78–1.18)	1.00 (0.80–1.24)
Right colon			
Men without prostate cancer	433/10,346 (4.2%)	Reference	Reference
Men with prostate cancer and ADT	13/320 (4.1%)	0.97 (0.55–1.70)	0.99 (0.55–1.79)
Men with prostate cancer without ADT	29/895 (3.2%)	0.77 (0.52–1.12)	0.73 (0.48–1.09)
Left colon			
Men without prostate cancer	480/8,175 (5.9%)	Reference	Reference
Men with prostate cancer and ADT	6/217 (2.8%)	0.46 (0.20–1.03)	0.52 (0.23–1.19)
Men with prostate cancer without ADT	37/602 (6.1%)	1.05 (0.74–1.48)	1.05 (0.73–1.51)
Rectum			
Men without prostate cancer	379/3,681 (10.3%)	Reference	Reference
Men with prostate cancer and ADT	3/63 (4.8%)	0.44 (0.14–1.40)	0.44 (0.14–1.43)
Men with prostate cancer without ADT	36/312 (11.5%)	1.14 (0.79–1.63)	1.20 (0.83–1.73)

*Note*: Mixed‐effects logistic regression was used to derive odds ratios (ORs) with 95% confidence intervals (CIs).

Abbreviation: ADT, androgen deprivation therapy.

^a^ With adjustment for age at diagnosis, Charlson comorbidity index, body mass index, educational attainment, and year of diagnosis.

In the main analysis, the unadjusted analysis demonstrated a decrease in anastomotic leakage among those exposed to ADT (OR 0.62, 95% CI 0.39–0.92) but not in men with prostate cancer without ADT exposure (OR 0.97, 95% CI 0.78–1.18) compared with men without prostate cancer. The adjusted analysis rendered an attenuated reduction for those exposed to ADT (OR 0.70; 95% CI: 0.45–1.09). Using tumour location as an interaction term, the apparent reduction in leakage was demonstrated for the left colon (OR 0.52, 95% CI 0.23–1.19) and the rectum (OR 0.44, 95% CI 0.14–1.40) but not for the right colon (OR 0.99, 95% CI 0.55–1.79).

The sensitivity analyses are summarized in Table 3. After adding prostatectomy and prostate irradiation as confounders, results were similar to the main analysis (OR 0.66, 95% CI 0.41–1.05). Using a more stringent definition of ADT exposure, results were again comparable (OR 0.72, 95% CI 0.46–1.13). The addition of ASA as a covariate rendered similar findings (OR 0.71, 95% CI 0.46–1.11).

**TABLE 3:** Sensitivity analyses for the main analysis of exposure to androgen deprivation therapy in relation to anastomotic leakage after colorectal cancer resection.

ADT group: sensitivity analyses	Adjusted^a^ OR (95% CI)
Prostatectomy/prostate irradiation as additional confounders	
Men without prostate cancer	Reference
Men with prostate cancer and ADT	0.66 (0.41–1.05)
Men with prostate cancer without ADT	0.97 (0.73–1.28)
Stringent definition of ADT exposure	
Men without prostate cancer	Reference
Men with prostate cancer and ADT	0.72 (0.46–1.13)
Men with prostate cancer without ADT	1.01 (0.82–1.26)
ASA fitness grade as additional confounder	
Men without prostate cancer	Reference
Men with prostate cancer and ADT	0.71 (0.46–1.11)
Men with prostate cancer without ADT	0.99 (0.79–1.23)

Abbreviations: ADT, androgen deprivation therapy; ASA, American Society of Anesthesiologists.

^a^ With base adjustment for age at diagnosis, Charlson comorbidity index, body mass index, educational attainment, year of diagnosis, and tumour location.

### Participants in the analysis of androgen receptor expression

Initially the substudy included a case group consisting of 45 patients who had experienced anastomotic leakage as well as a control group consisting of 46 patients who had not. In the end, 12 cases had to be excluded: six due to missing tissue samples and six due to insufficient quality and/or the presence of malignancy in tissue samples. The 33 remaining cases were randomly matched (1:1) to 33 of the 46 initially identified controls, with sex and type of surgery (right sided or left sided/subtotal) as matching criteria (Figure S1, Table S1). Tissue samples from 66 patients were therefore studied, and the scoring of the immunohistochemical staining was analysed as matched data.

### Androgen receptor expression

**FIGURE 4:**
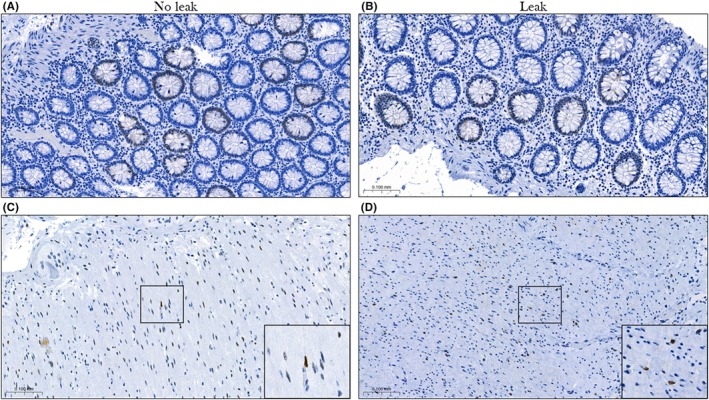
Representative examples showing the very low level of androgen receptor expression in the epithelium (A, B) and the muscularis propria (C, D) tissue layers of matched controls (A, C) and cases (B, D). No obvious difference in levels of expression can be observed.

The overall expression of androgen receptors in the colonic tissue of the case and control groups was low (Figure 4). The number of patients expressing some level of androgen receptor was slightly higher in the control group when compared with the case group (Table S2). This trend was observed for expression in both the epithelial and muscular layers. These differences between the groups were, however, not statistically significant (*p* = 0.092 and *p* = 0.118, respectively) (Table S3).

## DISCUSSION

In this nationwide study of male colorectal cancer patients, a potential but statistically nonsignificant reduction in risk of anastomotic leakage was detected as a function of present or recent ADT exposure in men with prostate cancer. The possible effect was seen for cancers in the left colon and rectum, while patients with right colon cancer were not affected. Prostate cancer controls without ADT exposure were no different from controls without prostate cancer in terms of anastomotic leakage, corroborating the notion that androgen exposure might play a role in the pathophysiology of leakage.

Differing rates of anastomotic leakage between men and women is a known phenomenon. Nevertheless, putative reasons to explain this have not been extensively investigated. The hypothesis that the wider pelvis in women enables easier dissection and therefore a lower risk of leakage has some support from recent pelvimetric data [4], but leak rates also differ after colon cancer operations [23]. Some experimental data suggest lower deposition of anastomotic collagen in male rats [7], while other studies indicate that differences in visceral fat distribution between the sexes might contribute to varying leak frequencies [5]. Even more pertinent to the present data, a study on laser resection of dog prostates revealed that androgen deprivation by surgical castration promoted wound healing, while testosterone supplementation prolonged the inflammatory phase [24]. Moreover, topical application of an androgen receptor antagonist on experimental cutaneous wounds in mice promoted wound healing through accelerated keratinocyte migration; the opposite effect was demonstrated using testosterone, where such migration in part was mediated through attenuation of transforming growth factor‐β and β‐catenin signalling [25]. Whether such mechanisms are important in the human intestine is a matter of speculation but could provide avenues for further research. In a previous study from our group, we were able to demonstrate that women with exposure to oestrogen‐increasing drugs had a lower rate of colorectal anastomotic leakage compared with female controls, again suggesting that hormonal influences might explain some of the differences seen between men and women [8].

Strengths of the nationwide registry‐based study include the population‐based design, mitigating selection bias. Missing data were rare and important confounders such as socioeconomic status, BMI and comorbidity were available. There are several weaknesses in the present study. There is likely to be some misclassification of both exposure and outcome. While the definition of exposure assumes that withdrawals equal drug intake it is not possible to know this for certain. This might be alleviated by excluding those with only one prescription withdrawal, as such a requirement would increase the chance of patients truly taking the drug. The restriction to patients undergoing curative colorectal cancer resection with anastomosis also increases the probability of stable disease in those with a history of prostate disease, since this may not have been the treatment of choice in a palliative setting. While the validity in general is high in the National Patient Registry [14], the actual misclassification for surgical castration, prostatectomy and prostate irradiation is not known. On the other hand, the known underreporting of leakage in the SCRCR might be a reason for the wide confidence intervals, as this nondifferential misclassification probably leads to attenuation of associations; this might be the chief reason why the adjusted estimates were not statistically significant. Moreover, despite careful inclusion of covariates based on a causal diagram there is a risk of residual confounding, as in all observational studies. Arguably the main limitation of the present study consists of the small number of those exposed to ADT, leading to few events of anastomotic leakage despite the nationwide study and large sample size overall.

In the experimental substudy, where androgen receptor expression in intestinal tissue was analysed, the overall expression was very low for both patients experiencing a leak (cases) and those without a leak (controls). A nonsignificant difference was observed between the cases and controls regarding the number of patients having some level of androgen receptor expression, with this number being higher in the control group. Although the implemented binary scoring was useful considering the low level of expression observed, it did lead to a low threshold for what was considered an androgen receptor‐expressing tissue sample. Findings using this scoring system might therefore lack clinical relevance, as they might exaggerate differences between patients that in fact were quite small. The findings in the substudy suggest that the trend of lower leak rates associated with decreased levels of androgen in patients exposed to ADT observed in the nationwide registry study is probably not directly related to androgen receptor expression in colonic tissue and could be mediated through effects beyond classical signalling through androgen receptor in the intestinal wall. Further research is required before any such conclusions can be drawn. It is important to note that rectal resections were not included in the substudy.

In conclusion, the nationwide registry‐based study has indicated that men with prostate cancer exposed to ADT might have a decreased risk of anastomotic leakage after colorectal cancer surgery. The finding must be considered tentative, as confidence intervals were wide, and residual confounding is certainly possible. The mechanism of the effect of androgen in intestinal tissue remains elusive as the expression of colonic androgen receptors was very low and showed no significant association with anastomotic leakage. Nevertheless, the present results are consistent with earlier experimental data on wound healing and other studies on sex‐related differences. Further research is warranted, as colorectal anastomotic leakage is a major clinical problem and the role of androgen modulation could be important.

## AUTHOR CONTRIBUTIONS

**Martin Rutegård:** Conceptualization; investigation; funding acquisition; writing – original draft; methodology; validation; writing – review and editing; project administration. **Isac Norrgård:** Investigation; writing – original draft; writing – review and editing; methodology; visualization; formal analysis; data curation. **John Moshtaghi‐Svensson:** Investigation; writing – review and editing; visualization; methodology; software; formal analysis; data curation. **Jaana Hagström:** Funding acquisition; writing – review and editing; investigation; validation; methodology; visualization; supervision. **Ida Hed Myrberg:** Writing – review and editing; resources; supervision; validation; methodology. **Anna Lantz:** Writing – review and editing; conceptualization; investigation. **Suvi Rasilainen:** Funding acquisition; writing – review and editing; supervision; validation; methodology. **Caroline Nordenvall:** Conceptualization; investigation; writing – review and editing; funding acquisition; resources; project administration. **Malin Sund:** Conceptualization; investigation; writing – review and editing; methodology; validation; supervision; project administration.

## FUNDING INFORMATION

Swedish Society of Medicine, the Stockholm Cancer Society, Swedish Cancer and Allergy Foundation, Region Västerbotten, Umeå University, Finska Läkaresällskapet and Medicinska Understödsföreningen Liv & Hälsa.

## CONFLICT OF INTEREST STATEMENT

None declared.

## ETHICS APPROVAL STATEMENT

The study was approved by the Regional Board of the Ethical Committee in Stockholm, Sweden (DNR: 2014/71‐31, 2018/328‐32, 2021‐00342, 2023‐03305‐02) and Helsinki, Finland (1223/2021).

## PERMISSION TO REPRODUCE MATERIAL FROM OTHER SOURCES

No material from other sources was included in this article.

## Supporting information


**Data S1** Supporting information.

## Data Availability

The data that support the findings of this study are available from Colorectal Cancer DataBase Sweden. Restrictions apply to the availability of these data, which were used under license for this study. Data are available from the author(s) with the permission of Colorectal Cancer DataBase Sweden.
