# Supplementary material for: Exposure to androgen deprivation therapy and risk of anastomotic leakage after colorectal cancer surgery
Source: Colorectal Dis. 2025 Jun 3;27(6):e70126. doi: 10.1111/codi.70126 (PMC12134439; doi:10.1111/codi.70126)
Supplement: Supplementary file 1 — Data S1 Supporting information. [file CODI-27-0-s001.docx]

Supplement

**Supplementary Table 1**. Descriptive statistics of the 66 participants in the analysis of

androgen receptor expression in intestinal tissue.

|  |  | Anastomotic Leak | |
| --- | --- | --- | --- |
|  |  | No | Yes |
| Categorical Variables | | N (%) | N (%) |
| Sex | Male | 20 (60.6) | 20 (60.6) |
|  | Female | 13 (39.4) | 13 (39.4) |
| Surgery | Right sided | 10 (30.3) | 10 (30.3) |
|  | Left sided or subtotal* | 23 (69.7) | 23 (69.7) |
| Diagnosis^#^ | Cancer | 22 (66.7) | 18 (54.5) |
|  | Benign | 11 (33.3) | 15 (45.5) |
| Continuous Variables | | Median (IQR) | Median (IQR) |
| Age (years) |  | 73 (63–77) | 74 (64–80) |

**A total of three patients had a subtotal colectomy. Considering the similarities in leak rates between subtotal and left sided*

*colectomies (21), the patients were included in the same group as the patients who had a left sided colectomy.*

*# Diagnosis refers to primary disease leading to need of colorectal surgery.*

IQR = interquartile range

**Supplementary Table 2.** *Expression of androgen receptors (AR) in the control group and the case group.*

|  |  | Anastomotic Leak | |
| --- | --- | --- | --- |
|  |  | No | Yes |
| Expression of AR: |  | N (%) | N (%) |
| Epithelium | Negative | 12 (36.4) | 19 (57.6) |
|  | Positive | 21 (63.6) | 14 (42.4) |
| Muscularis propria | Negative | 11 (33.3) | 18 (54.5) |
|  | Positive | 22 (66.7) | 15 (45.5) |

**Supplementary Table 3**. *The presence or absence of androgen receptor expression in the colonic tissue samples of 33 pairs of matched cases and controls. These show the number of pairs without any androgen receptor expression in their tissues (a), pairs where expression was found in both the control and case (d), and pairs with expression in either the control or case (b, c).*

| **Expression in the epithelium** | | |
| --- | --- | --- |
| Expression in cases | Expression in controls | |
|  | Negative | Positive |
| Negative | 9 (a) | 10 (b) |
| Positive | 3 (c) | 11 (d) |

| **Expression in the muscularis propria** | | |
| --- | --- | --- |
| Expression in cases | Expression in controls | |
|  | Negative | Positive |
| Negative | 7 (a) | 11 (b) |
| Positive | 4 (c) | 11 (d) |


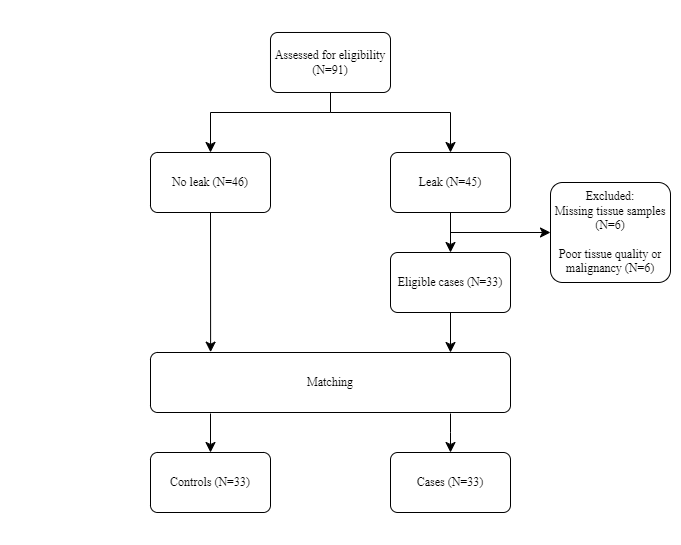


**Supplementary Figure 1**. Flowchart depicting the process of participant exclusion and matching for the sub study of androgen receptor expression in colonic tissue.
